# Thermophysical Properties of Temperature-Sensitive Paint

**DOI:** 10.3390/ma14082035

**Published:** 2021-04-18

**Authors:** Andrzej J. Panas, Robert Szczepaniak, Wit Stryczniewicz, Łukasz Omen

**Affiliations:** 1 Faculty of Mechatronics, Armament and Aerospace, Military University of Technology, Gen. S. Kaliskiego Street No 2, 00-908 Warsaw, Poland; lukasz.omen@wat.edu.pl; 2 Faculty of Aeronautics, Military University of Aviation, Dywizjonu 303 Street No 35, 08-521 Deblin, Poland; r.szczepaniak@law.mil.pl; 3 Aerodynamics Department, Łukasiewicz Research Network—Institute of Aviation, Krakowska Street No 110/114, 02-256 Warsaw, Poland; Wit.Stryczniewicz@ilot.lukasiewicz.gov.pl

**Keywords:** temperature-sensitive paint, complex thermophysical study, thermal diffusivity, heat capacity, thermal conductivity, thin layer properties, inverse analysis

## Abstract

The complex thermophysical property of temperature-sensitive paint (TSP) research is discussed. TSP is used for visualization of the surface temperature distribution in wind tunnel aerodynamic tests. The purpose of this research was to provide reliable, experimental, thermophysical data of the paint applied as a coating. As TSP is applied as thin surface layers, investigation of its final properties is challenging and demands the application of non-standard procedures. At present, most measurements were performed on composite specimens of TSP deposed onto a thin metallic film substrate or on TSP combined with a cellulose sheet support. The studies involved gravimetric,, thermogravimetric, and microcalorimetric analyses, transversal thermal diffusivity estimation from laser flash data and in-plane effective thermal diffusivity measurements done by the temperature oscillation technique. These results were complemented with scanning electron microcopy analysis, surface characterization and the results of dilatometric measurements performed on the TSP bulk specimens obtained from liquid substrate by casting. Complex analysis of the obtained results indicated an isotropic characteristic of the thermal diffusivity of the TSP layer and provided reliable data on all measured thermophysical parameters—they were revealed to be typical for insulators. Further to presenting these data, the paper, in brief, presents the applied investigation procedures.

## 1. Introduction

Temperature-sensitive paint (TSP) provides a high spatial resolution of surface temperature measurements. This makes TSP suitable for the investigation of complex flows in wind tunnels, such as boundary layer transition and shock/boundary-layer interaction [1,2]. The easeof coating deposition and short response time of the TSP technique allow for measurements in relevant industrial applications such as film cooling [3,4] and heat transfer studies in high-speed facilities [5,6,7]. The TSP technique, along with temperature-sensitive coatings such as thermographic phosphors [8,9] and thermographic liquid crystals [10], has become an attractive alternative to well-established but invasive thermocouple measurements and IR thermography [11], in some cases being sensitive to changes in experimental conditions [12]. Although the temperature-sensing technique in TSP is similar to, e.g., luminescence sensing of a thermal load inthermal barrier coatings [13,14], application of aerosol-sprayed thin TSP layers in wind tunnel applications creates completely different challenges for their use, mostly because of the usually much lower temperature differences needed for detection. Although the high spatial and temporal resolutions of TSP data make this technique suitable for detailed heat flux studies in wind tunnels, application of an additional layer on a metallic model might impede the investigated heat transfer process [15]. In the case of heat transfer investigations based on the determination of global surface temperature distribution, the thermal properties of all layers should be included in the analysis. This creates the need to know the thermophysical properties of the TSP layer and its dependence on the layer structure. Moreover, the complex set of thematic parameters, including density, heat capacity and thermal conductivity, must be known as accurately as possible when considering transient heat transfer phenomena.

In TSP, luminescent particles are used to detect temperature changes, such that when the particles are excited with light of a certain wavelength, light of a longer wavelength is emitted [2]. Both the emission wavelength and the excitation duration depend on the actual temperature of the structure containing the luminescent particles. Luminescent particles are only an additive to the dispersed composite structure of the TSP layer with a polymer binder and pigments filling, usually titanium dioxide. The last two components dominate. Their actual proportions are a secret of the manufacturer, but it is known that the paint spraying fluid is based on benzotrifluoride at a content of approximately 90% by volume. The properties of individual components, including thermophysical data, are usually known. The problem with predicting the properties of the target TSP layer does not arise from the introduction of new the more sensitive substances [16] or binders [17]. The layer structure is the most problematic as the properties of a deposited film might significantly differ, both from the liquid mixture and from individual solid components [18]. Cai, in [15], arbitrarily assumed, for a TSP layer’s thermophysical properties, the same properties applied for Mylar foil that resulted in a room-temperature thermal conductivity of *λ* = 0.15 W·m^−1^·K^−1^, a density of *ρ* = 1300 kg·m^−3^ and a specific heat of *c_p_* = 1090 J·kg^−1^·K^−1^. The reliability of Cai’s assumption was debatable and required verification. While analyzing TSP thermophysical properties, their dependence on temperature needs to be accounted for. According to the results of Peng at al. [19] from hypersonic tunnel experiments, the temperature increase Δ*T* can reachup to 80 K. Liu at al. [20] studied the effect of temperature-dependent diffusivity on investigated TSP heat flux measurements, but in the set of considered materials, the TSP layer was not included, most probably because of a lack of necessary data.

This paper presents the results of an investigation of the detailed complex thermophysical properties of TSP layer material. The initial research results, based on not fully verified data, were reported in the conference paper [21]. Basic measurements were performed for a thin layer coating deposited by aerosol spraying in the same way that objects investigated in a wind tunnel are covered. The investigated coating was applied on metallic foil substrate in order to properly reflect the structure and properties of real sensing paint. Depending on the investigation type, different foils were applied as substrates: thicker molybdenum foil of about 0.1 mm thickness and about ten times thinner aluminum foil. Utilization of such a mechanical support was necessary due to problems with performing measurements on a free-standing layer with thicknesses recommended for temperature sensing. This also allowed to simulate the most common conditions of thermal contact between a layer and substrate, as TSP is typically applied on metal models in wind tunnel testing. The TSP density and moisture absorption properties were determined using gravimetric and thermogravimetric (TG) measurements. Simultaneously, the paint layer surface and cross-section were investigated with use of scanning electron microscopy (SEM). The specific heat temperature characteristic was determined with the use of differential scanning calorimetry (DSC). The set of thermophysical properties data of the TSP was completed with thermal conductivity (TC) data determined with the use of an in-house inverse procedure for the determination of thin layer TC from laser flash thermal diffusivity (TD) measurements [22,23]. By applying this methodology, the out-of-plane TC/TD of the investigated layer can be obtained. In spite of the fact that there had not been any indications of anisotropy of the analyzed TSP structure, additional measurements of the in-plane TD were made on a specially prepared composite TSP paper specimen. The temperature oscillation technique was used for the in-plane thermal diffusivity investigation [12]. As the applied technique, in the case of a composite specimen investigation, needs a low thermal conductivity, support composite specimens for these investigations were prepared by repeatedly soaking and drying paper strips. However, it turned out that properties of a relatively thick TSP layer formed differently than the properties of an aerosol-deposited TSP layer. Nevertheless, the test results are included in this report as additional data characterizing the tested material; the obtained results complement the results of the TC/TD investigation from the inverse procedure. For additional measurements, a dilatometric study was also performed on a sample manufactured by TSP liquid substrate casting–drying in a silicon mold 12.5 mm in diameter and about 2 mm in height.

## 2. Experiments

The temperature-sensitive paint under investigation was UniTemp TSP supplied by the manufacturer Innovative Scientific Solutions Inc. (ISSI, Dayton, OH, USA). The exact chemical composition of the TSP was unknown. The substrate for airbrush spraying or for painting the TSP films was delivered as a fluid. Generally, the TSP layers were applied by aerosol spraying onto metallic foils in three and five passes of the airbrush. Between subsequent passes, the specimens were allowed to evaporate the liquid solvent. Repetition of airbrushing operation was necessary to obtain uniform TSP covering. The typical thickness of such a layer is several dozen micrometers. The small thickness and brittleness of the structure make it difficult not only to test but also to prepare it as a free-standing specimen. For this reason, metallic foils imitating the substrate from TSP sensing applications were utilized to support the tested layers. For laser flash out-of-plane TD, stiff molybdenum foil was applied for measuring TSP coatings [18,24]. In the course of measurement, two type of specimens were investigated, i.e., an approximately 15-μm thick layer deposited using three airbrush passes and an approximately30-μm thick layer deposited in five passes. For TG and DSC investigations, the TSP was airbrushed on thin aluminum foil in order to achieve a higher in proportion amount of the paint material on the manufactured structure. In all cases, the TSP was airbrushed manually from a distance of about 100 mm.

In addition to studies of a TSP layer prepared by aerosol spraying, additional measurements were performed on specimens prepared by repeated drying of thin layers of liquid substrate poured into a mold or on a paper backing. All specimens were prepared and dried at room temperature.

## 3. Materials and Specimen Preparation

For the basic thermophysical property (TP) measurements, two types of substrates were used: (i) aluminum foil of a thickness of 11.1 µm and (ii) molybdenum sheets of a thickness of 96 µm. The aluminum foil was utilized for specimen preparation for gravimetric, TG and DSC measurements. Molybdenum was selected as a TSP layer substrate for laser flash apparatus (LFA) analysis [25] investigations due to the relatively high TD of molybdenum and sufficientfoil stiffness for carrying TSP layers at LFA sample holders [24]. The complementary temperature oscillation TD measurements were performedon a TSP structure developed on a sheet of paper of 0.1 mm thickness. Paper was selected forreinforcement of the investigated TSP–paper composite structure because of its low TC. Such low TC reinforcement would not affect the in-plane TD measurement much. 

For the LFA experiments, two composite specimens were prepared. The disc-shaped molybdenum substrate was 12.5 mm in diameter and 96 µm in thickness. The TSP layers were applied on one side of the molybdenum substrate. Before LFA investigations, both sides of the specimens were covered with flake graphite to improve the laser flash energy absorption on the molybdenum side and to improve the sample thermal response IR recording on the TSP side. An aerosol-sprayed GRAPHIT33, (KONTAKT CHEMIE, CRC Industries Deutschland GmbH, Iffezheim, Germany) flake paint preparation was utilized. Thin graphite layers, approximately 10 µm, were deposited [18,21]. As a result, four-layer structures of GRAPHIT33–molybdenum foil–TSP layer–GRAPHIT33 were investigated.

**Figure 1:**
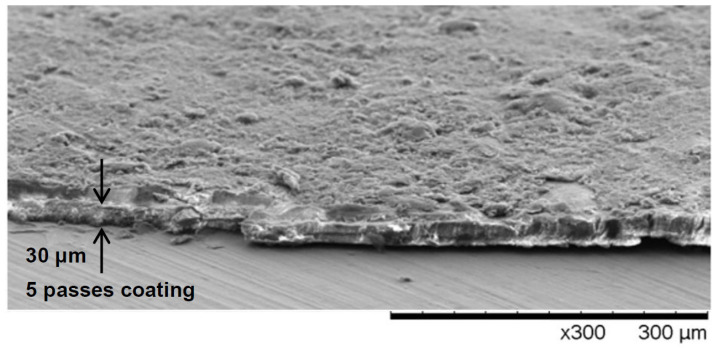
Scanning electron microscopy (SEM) image of temperature-sensitive paint (TSP) layer on molybdenum substrate after a layer fragment separation for microscopic structure inspection.

As recommended by the manufacturer, a single layer of TSP was applied in six small cross-layers of the airbrush. Parallel to the preparation of multilayer TSP structures, reference samples were prepared for microscopic examination of the structure of the coating cross-section, thickness uniformity and surface topography. In Figure 1, a typical result of the SEM investigation is shown. Analysis of the obtained SEM images indicated low layer porosity and structure uniformity. As for the thickness distribution, the three-pass reference coating thickness varied from 15 to 26 micrometers, while the five-pass reference coating had a relatively uniform thickness ranging from 27 to 32 micrometers. Based on the density and geometric data, the effective thicknesses of the three- and five-layer specimens tested during the thermophysical measurements were calculated. The respective values were assumed to be equal to 15 and 30 micrometers, respectively (Figure 1).

**Figure 2:**
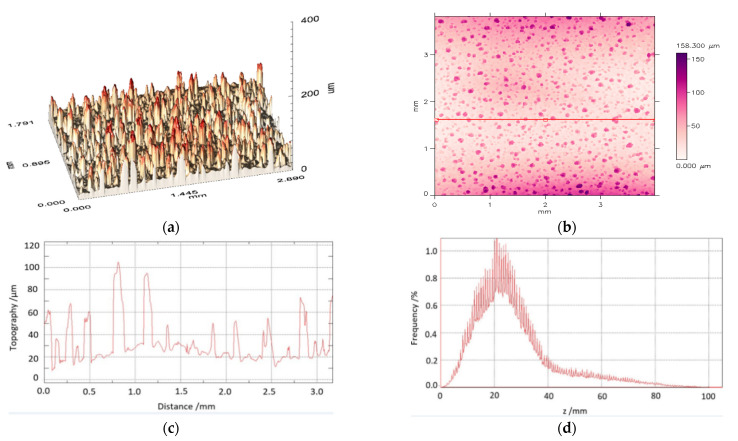
Results of the TSP coating surface morphology investigation: (**a**) Three-dimensional surface image; (**b**) two-dimensional surface mapping; (**c**) typical profile (Figure 2b, red line cross-section); (**d**) diagram of a normalized dimensional spectral analysis result.

The TSP layer surface roughness was investigated and characterized using 2D and 3D surface scanning by applying an optical MicroProf 100 profilometer, FRT GmbH a FORMFACTOR company, Cologne, Germany. The 3D surface morphology profile is shown in Figure 2a. Figure 2b show the surface mapping results obtained for a typical profile selected for data processing from around a dozen chosen by random. The surface profile along the red line from Figure 2b is illustrated in Figure 2c, while the result of the profile spectral analysis is depicted in Figure 2d. The spectrum average is equal to 21 μm, and 80% of the data points are comprised within an interval of ±12 μm around this value. The numerical parameters of the TSP coating surface morphology study for the selected profile are presented in Table 1.

**Figure 3:**
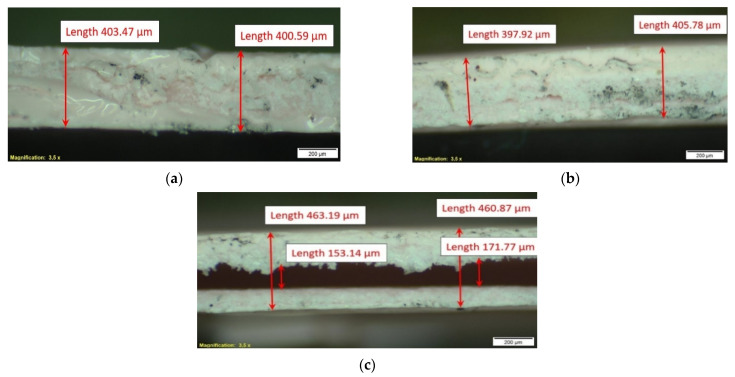
Microstructure of TSP–paper composite specimens: cross-section of one longitudinal cut of specimen A (**a**) and two parallel longitudinal cuts of specimen B (**b**) thickest section; (**c**) thinnest section with a longitudinal split cave).

Specimens for the temperature oscillation TD measurements were prepared by repeatedly soaking and drying strips of paper once folded. Such a procedure allows to fill the internal pores of paper with the TSP as much as possible. The paper strips prepared for repeated soaking and drying were 210 mm in length and 20 mm in width. The strip thickness was around 0.1 mm. Prior to investigations, these strips were transversely cut into segments of 20 mm in length, about 5 mm in width and about 400 μm in thickness. The last number includes around a 200-μm thickness of two paper sheets from inside. The specimens’ microstructure examination was performed after accomplishing the temperature oscillation in-plane TD measurements; selected results are presented in Figure 3. The examination revealed longitudinal separation within specimen B at about half of its length (Figure 3c). As for the longitudinal (in-plane) propagation of the temperature oscillation, it should not significantly affect the measurement result but should be taken into account when analyzing the result data.

## 4. Weightings and Thermogravimetric Measurements

Gravimetric measurements were performed with use of an AT261 Delta Range, Mettler-Toledo, Greifensee, Switzerland, analytical microbalance. The device resolution was 0.01 mg. The mass of the samples was measured after the layer deposition. The density of samples was determined with the use of a balance equipped with a Density Kit using the buoyancy technique.

The densities of aluminum and molybdenum foils, the substrates for aerosol spraying deposition of TSP, were determined using buoyancy weightings [18,21]. The masses of TSP layers were determined by subtraction of the substrate mass, measured before TSP spraying, from the mass of the final composite specimen. Two types of density investigations were performed depending on the type of TSP sample. The TSP coating layer density was determined using an indirect method as described in [18]; in buoyancy measurements, the density of the aluminum foil substrate and the effective density of the TSP layer–aluminum foil sandwich were first determined independently, and then, the TSP layer density was calculated. The density of the TSP coating deposited by aerosol spraying was determined to be 1980 ± 260 kg·m^−3^. The effective density of the TSP–paper composite and the density of the TSP specimens manufactured by bulk deposition and drying/casting were determined by buoyancy weighting. The TSP bulk specimen density was determined to be 1375 ± 35 kg·m^−3^. The effective densities of two TSP–paper composite specimens were equal to 1430 kg·m^−3^ for the specimen indicated as A and 1410 kg·m^−3^ for specimen B. Considering the lack of pores indicated in the SEM characterization of the TSP–paper structure, the last values can be treated as valid for TSP material filling strictly fibrous cellulose structures. Helium pycnometry measurement of cellulose fibers’ density resulted in a density value of 1700 kg·m^−3^. The results of the TSP–paper structure, falling between the casted TSP density and the density of cellulose fibers, suggest differences in the final structure or composition between the TSP structure deposited by aerosol spraying and that deposited from the liquid bulk.

Using TSP–paper specimens’ weightings data, the cellulose fibers’ density and the effective density value of a paper sheet equal to 780 kg·m^−3^, the cellulose volumetric share was calculated as 19 vol.% for specimen A and 31 vol.% for specimen B. The expected densities of specimens A and B, calculated by applying these data, were equal to 1437 and 1477 kg·m^−3^, respectively. The calculated values are within the uncertainty limits of the aforementioned density measurement results and properly reflect differences in the composition of the TSP–paper composites A and B. 

**Figure 4:**
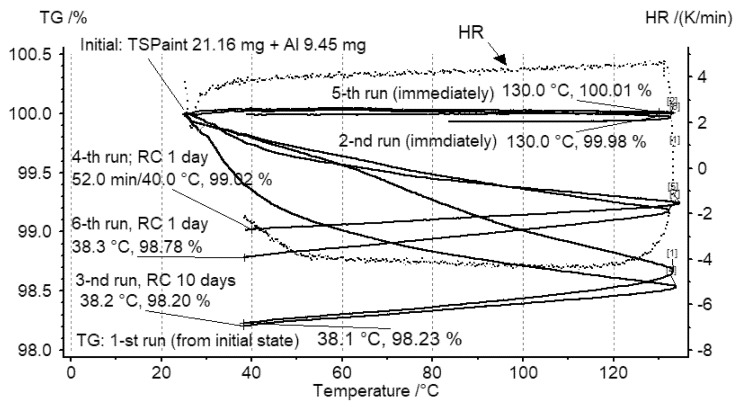
Results of thermogravimetric (TG) investigation of TSP deposited by aerosol spraying on aluminum foil substrate.

TG investigations were performed by utilizing a Thermo Microbalance TG 209 F3 Tarsus, NETZSCH Gerätebau GmbH, Selb, Germany, thermobalance under inert gas Argon atmosphere at a flow rate of40 mL·min^−1^. During TG studies, both the remaining solvent effects and the thermal stability of the TSP layer materials were checked. Systematic TG studies were performed for the thin layer TSP–aluminum foil specimen. The sample temperature was increased from approx. 25 to 130 °C and cooled back to room temperature in two consecutive heating programs. The first heating revealed a mass loss of 0.54 mg in the TSP–aluminum foil sample that is equivalent to 2.55% TSP layer mass loss, whereas in the second heating, the loss of mass was negligible (Figure 4). The measurements were repeated after 10 days (3rd run) and on two consecutive days (4th, 5th and 6th runs) in order to investigate the influence of moisture absorption of the TSP. The measurements performed after 1 day of specimen seasoning revealed lower mass losses at experiment repetition. The final mass loss at the 6th run was equal to 0.37 mg. It resulted in 1.78% mass loss when referring to the TSP mass only. A comparative plot of all runs is presented in Figure 4. The TSP mass loss in the 3rd run was almost the same as that in the 1st run; there were no distinct mass losses in immediately repeated measurements (2nd and 5th runs) and the mass losses after one day of specimen seasoning were about 1.5% (4th and 6th runs). Additional measurements performed for the casted TSP–paper structure resulted in similar results, confirming the moderate sensitivity of the paint to humidity at the laboratory scale.

## 5. Dilatometric Measurements

**Figure 5:**
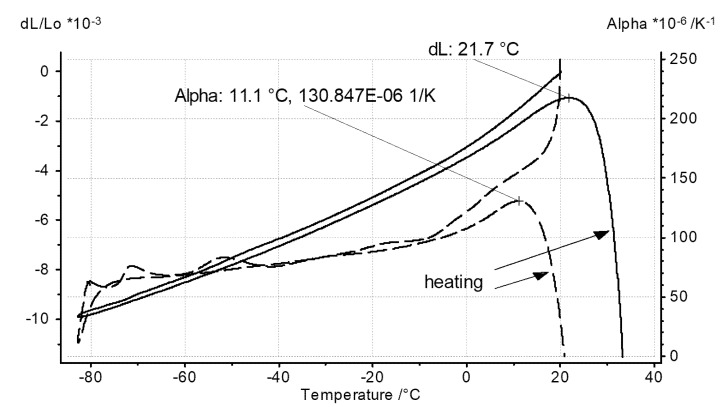
Results of dilatometric investigation of the TSP bulk specimens manufactured by drying the liquid substrate poured into a mold (solid line—linear expansion; dashedline—coefficient of linear thermal expansion).

Dilatometric measurement was performed on a specimen cut from a disc of 12.5 mm in diameter prepared by drying of a liquid TSP substrate poured into a silicon mold. The sample of this material was used for density measurements. During this study, linear expansion was measured utilizing a DIL 402 C, NETZSCH Gerätebau GmbH, Selb, Germany, pushrod dilatometer [26]. The system was equipped with a liquid-nitrogen-cooled furnace, allowing measurements from −180 up to 500 °C. The test had been planned to be conducted between −80 and 130 °C on thermal cycling and was started from 20 °C at cooling to −80 °C segment but was finally stopped at about 30 °C due to specimen collapse upon material softening. The experiment was not repeated as the specimen still showed plastic behavior at a temperature of approx. 35 °C.For the discussed investigations, a fused silica specimen holder and pushrod, which had previously been calibrated against a 12-mm sapphire reference, was employed. The test was carried out in a static inert helium atmosphere at a heating/cooling rate of 4 K·min^−1^. The coefficient of linear thermal expansion referring to the initial specimen length (linear thermal expansivity/coefficient of linear thermal expansion (CLTE)/physical alpha) was derived from the measured thermal expansion by applying a standard Proteus, NETZSCH Gerätebau GmbH, Selb, Germany, software. The results shown in Figure 5 indicate a kind of glass transition with onset between −5 and 0 °C. The CLTE values of the investigated bulk TSP specimen are typical for polymers [27].

## 6. Microcalorimetric Measurements

**Figure 6:**
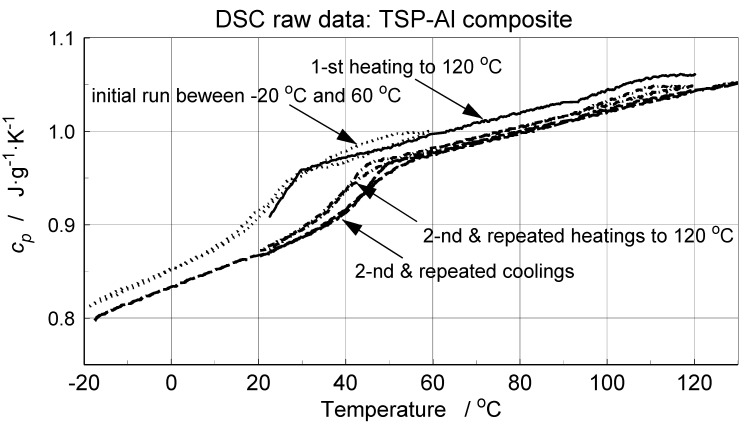
Direct results, not corrected for mass changes, of microcalorimetric investigation of TSP deposited by aerosol spraying on the aluminum foil substrate—the effective heat capacity in function of the temperature.

Microcalorimetric measurements were performed usinga power-compensated Pyris 1 DSC, PerkinElmer, Inc., Waltham, MA, USA, microcalorimeter. The analysis was focused on determination of the specific heat using a standard three-curve procedure and a dedicated temperature program [28]. The temperature program was composed of heating and cooling steps, separated with isothermal periods. This procedure ensures the exact determination of the specific heat from both heating and cooling processes. The temperature range of the DSC measurements was from −20 to 120°C [28]. In order to identify any possible phase change and moisture absorption effects, the scans were repeated in subsequent step-scanning cycles [28]. Direct results of DSC data processingin the form of the effective specific sample heat dependence on temperature are presented in Figure 6. The effects of evaporation of residual solvent or moisture cause the difference between the first heating and all subsequent effective *c_p_* data for cooling and heating. A characteristic increase in the specific heat around 50 °C could likely be attributed to glass transition of a polymer TSP layer base.

**Figure 7:**
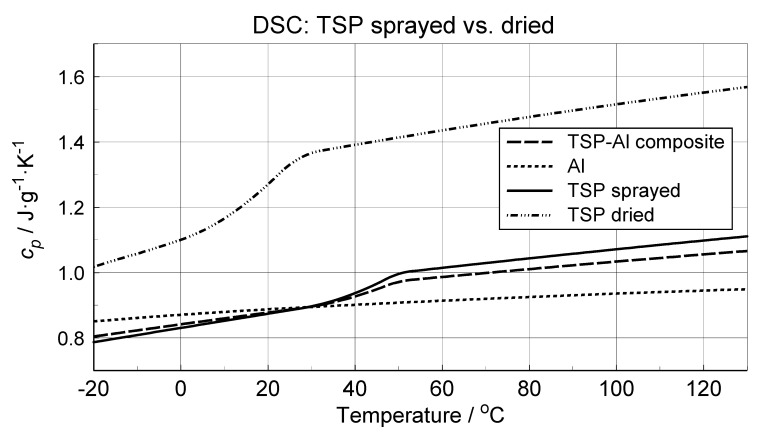
Processed results of microcalorimetric investigation of the TSP specific heat (TSP layer from aerosol spraying) with presentation of intermediate steps of the differential scanning calorimetry (DSC) measurement data approximation and the approximated data of the TSP structure obtained by the drying method.

The DSC data from repeated heating and cooling were smoothed using the B-spline approximation procedure [29]. The specific heat of the TSP component was determined indirectly from the DSC signal with the use of mass fractions of the composite sample components and the aluminum foil DSC study data. The *c_p_* values of TSP, aluminum foil and TSP–aluminum foil samples (in this instance, an effective *c_p_*, i.e., a composite specimen heat capacity) are presented in Figure 7. The aluminum characteristic closely matches the literature data. The two other characteristics indicate a glass transition occurring within the interval from about 20 to about 50 °C. 

A TSP layer obtainusing a drying method was investigated without any supporting structures. During the DSC study, the same procedures of measurement and subsequent data processing were applied. The obtained result is presented in Figure 7. The greater specific heat values with reference to the TSP aerosol layer suggest that a greater share of fluorohydrocarbon or copolymer, probably, remains in the casted TSP sample. Interestingly, the glass transitions of DSC data closely match, regarding the transition onset and end, the DSC data of the aerosol TSP layer from the first heating (see Figure 6). The approximate 10°C value of onset temperature is almost the same as the collapse temperature of the dilatometric specimen of the dried TSP (see Figure 5).

## 7. LFA Thermal Diffusivity Measurements

**Figure 8:**
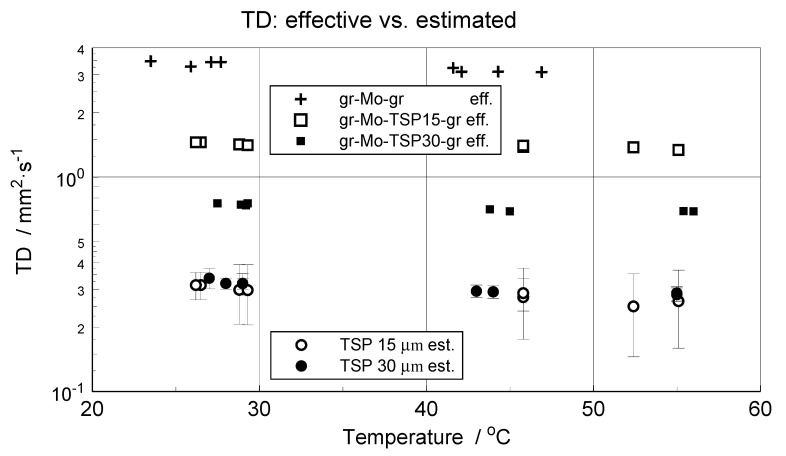
Apparent thermal diffusivity (TD) of the investigated composite GRAPHIT33–molybdenum–TSP–GRAPHIT33 specimens.

TD measurements were performed by applying a planar pulse heating method using a LFA 457 Microflash, NETZSCH Gerätebau GmbH, Selb, Germany [24,25]. The temperature range of the measurements was limited to the interval from 25to approximately 50 °C at doubled shots performed at 25, 30, 40and 50 °C. The specimens were pulse-heated from the substrate side covered with flake graphite. The thermal response, i.e., the course of temperature change over time, was recorded with an infrared detector from the top layer of TSP, also covered with a film of flake graphite. The apparent TD of a composite specimen was determined using Proteus LFA, NETZSCH Gerätebau GmbH, Selb, Germany. A variety of models were applied for TD calculations, and the Cowan model with pulse correction was chosen as the best to fit the experimental data, with a maximum error equal to 0.0145 and 0.00454 mm^2^·s^−1^ for 15 and 30 μm TSP coatings, respectively. Direct results of the apparent TD calculation obtained for GRAPHIT33–molybdenum–TSP–GRAPHIT33 specimens are presented in Figure 8. Maximum TD values of the composite specimens set the upper level of the TD of the paint for a given layer thickness. 

## 8. Estimation of the TSP Layer Thermal Diffusivity

As the thermal diffusivity of composite specimens does not comply with any mixing rules, even in the case of simple geometrical configurations of the components, the true TD of a certain component cannot be calculated just by subtraction. In order to retrieve the TD from a specimen, thermal response methods of inverse problem solving can be applied [30]. In the described case, a dedicated inverse parameter estimation procedure was applied—the recorded thermal response signal was fitted with a function representing a model response [23,31]. The procedure involved a direct heat transfer problem solution to obtain the temperature change of a four-layer sample exposed to short impulse heating imposed on the surface of the first layer. The temperature signal of the fourth layer’s surface was used in the estimation procedure. A 1D time-dependent heat conduction equation was solved utilizing the finite difference method and the finite element method. The thermal properties of the layers are listed in Table 1. The DSC measurements revealed a step increase in TSP specific heat (see Figure 7) within the experimental temperature range. This change was included in the thermal properties of the numerical model (see Table 2).

In the finite difference approach to solution of the 1D time-dependent heat conduction equation, a backward difference scheme for the time derivative and a central difference scheme for the space derivative were used. A boundary condition of step heat flux transfer on the surface of the first graphite layer was set. The time duration of the heat flux step was set to 0.6 ms in order to simulate the experimental laser pulse duration. A matching temperature condition on the interface of the layers was set, and no thermal contact resistance was included in the heat conduction equations. Convective heat transfer on both graphite surfaces, i.e., first and fourth layers, was imposed. The model was validated by determination of the TD of a domain composed of four layers of the same material. A difference of 0.4% was found between the TD determined using the Parker method [32] from the model thermal response and determined from the TP of the material. The number of mesh elements providing a difference of less than 1% was determined for the estimation procedure. Consequently, mesh dissection in the spatial domain was set to 400, 133 and 44 elements in the molybdenum, TSP and graphite layers, respectively. The time step *dt* and duration of the simulation *t_s_* were established from the experimental signal. Typical values of *dt* and *t_s_* were 0.05 and 20 ms, respectively. 

**Figure 9:**
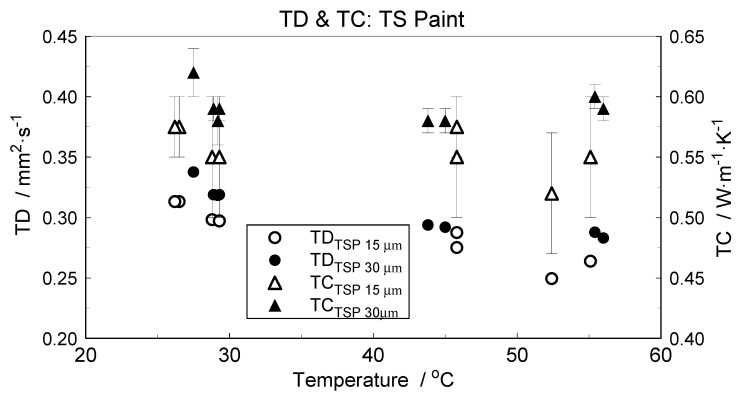
Estimated TSP thermal diffusivity (TD) and thermal conductivity (TC) of TSP.

For a known *ρ* and *c_p_* and estimated TD of the investigated TSP, its TC can also be determined. The estimated values of TSP thermal diffusivity and thermal conductivity are presented in Figure 9.

## 9. In-Plane Thermal Diffusivity Measurements

As discussed above, both the microscopic examination and the gravimetric characterization revealed distinct differences between the TSP layer from aerosol spraying and the bulk TSP–paper composite structure. The additional measurements of the in-plane TD of the TSP–paper structure could only provide complementary information about the thermal transport properties of the investigated paint. However, the information is valuable for overall evaluation of the thermophysical properties, not only in qualitative but also in quantitative aspects. 

**Figure 10:**
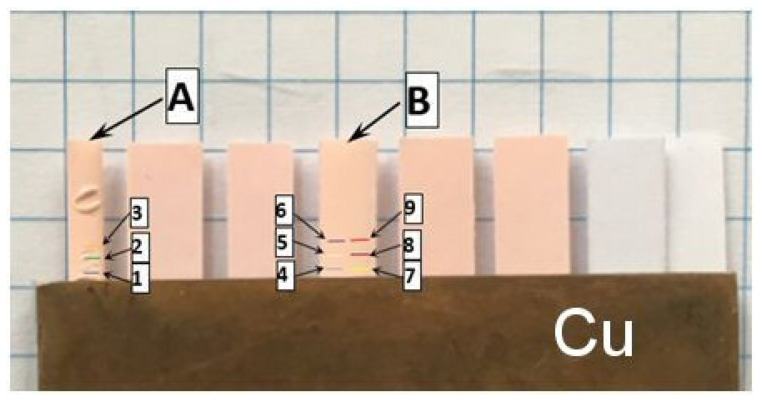
View of specimens prepared for infrared TD measurement by temperature oscillation with indication of control lines on the investigated TSP–paper composite specimens.

**Figure 11:**
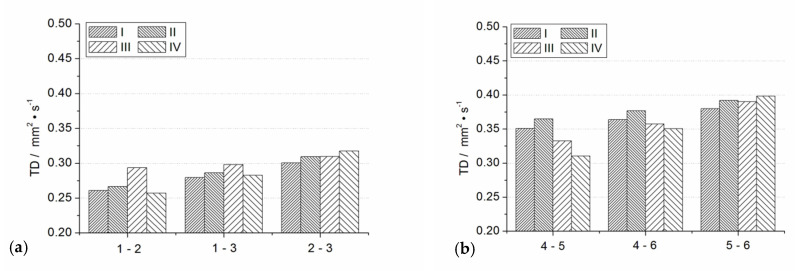

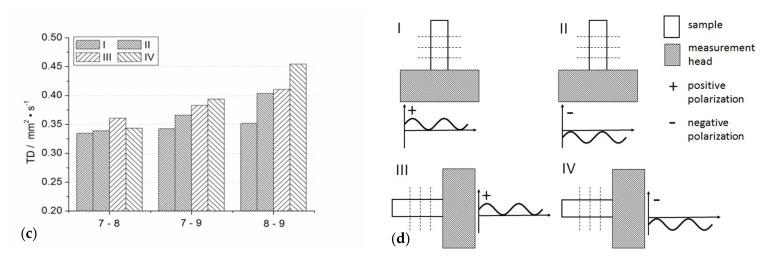
Results of in-plane TD measurements performed on composite specimens A (**a**) and B (**b**,**c**) compared with specimens’ arrangement in Figure 10; measurement configurations (**d**).

The in-plane TD measurements were performed by applying a temperature oscillation technique. Details concerning modifications introduced into Ångström’s classical method and a brief description of the experimental stand and the methodology of processing of the data from infrared temperature measurements are provided in [12]. The two measured TSP–paper specimens, indicated as specimen A and specimen B, were cut to the form of strips 20 mm in length with widths of 2.45 and 4.40 mm, respectively. The specimens were clamped between copper plates, being in direct contact with the Peltier elements of the measuring system, as shown in Figure 10. The applied temperature oscillation was of a period equal to 60 s and of an amplitude not exceeding 1 K. Measurements were performed for two configurations of the measuring head: with horizontal specimen alignment and vertical oscillation propagation (Figure 11a, cases I and II), and with vertical specimen alignment and horizontal oscillation propagation (Figure 11a; cases III and IV).Moreover, the tests were performed for two polarities of temperature oscillation excitation: positive, i.e., pulsating upwards (Figure 11a, cases I and III), and negative (Figure 11a, cases II and IV). For final processing, temperature recordings of 10 subsequent oscillation periods from lines separated at distances of 1.31 mm were taken (Figure 10, lines 1–3 at specimen A and lines 4–6 and 7–9 at specimen B). The TD values were calculated for all three possible combinations of temperature signals. The averaged results over the ten periods are shown in Figure 11. Standard deviations of the bars represented in Figure 11 are within the interval from 0.01 to 0.05 mm^2^·s^−1^, which is not greater than about 15% of the total average of the measured TD equal to 0.34 mm^2^·s^−1^. 

Surprisingly, this value matched, very well, the out-of-plane TD of the TSP layer estimated from LFA measurements (Figure 9). However, while comparing the results, one should remember the differences in structure between the TSP aerosol coating and the TSP–paper composite and also the revealed difference in density between the TSP coating deposited by spraying (1980kg·m^−3^) and the bulk TSP produced by soaking and drying (about 1400 kg·m^−3^). The obtained results of in-plane TD qualitatively justify the results of the TSP coating TD estimation. They also complete the overall characteristics of TSP structures. 

## 10. Discussion

In the global evaluation of the results obtained, attention will be focused on the main subject of research, i.e., on the airbrushed TSP layer material representing the same structure as temperature-sensing coverings applied in wind tunnels. Due to relatively low thickness and brittleness, investigations of a free-standing TSP specimen are extremely difficult. This had already been demonstarted by preliminary studies described in brief in [21]. Due to such metrological limitations, most conventional methods or procedures are excluded from use. Nevertheless, the application of dedicated procedures based on estimation of the layer material properties from the results of effective (or apparent) thermophysical property investigation of the studied thin films deposited on metallic substrates allows forobtaining reliable data of the measured parameters [23]. In this instance, the density, mass heat capacity and thermal conductivity values were extracted from the results of measurements performed on composite specimens. A side effect of this isrelatively large measurement errors. In the case of TD/TC, individual experiment inaccuracies exceed 35% in relative values (Figure 8 and Figure 9). In view of the investigation’s poor metrological conditioning, these values can be accepted—credibility is more important than precision.

The obtained data significantly differ, as shown in Table 3, from the data presented by Cai at al.and confirmed Cai’s assumption of similar TP values for Mylar and TSP to be incorrect [15]. It should be pointed out that Cai and coworkers applied Mylar thermophysical data for modeling the TSP layer properties. Our measured values of TD/TC are more than three times higher in comparison to those in [15]. The experiments performed indicate that the thermal contact resistance between the substrate and the TSP layer was negligible;this could be inferred from agreement of the TD/TC results obtained for two different layer thicknesses (Figure 9). Our 50% greater density values and 20% lower heat capacity values in reference to the data from [15] substantially differ from values typical for polymers. The additional measurements performed on casted TSP specimens indicate that TPs can vary greatly depending on how the sample is prepared. The scale of possible differences is illustrated by the data presented in Table 2. As the results from the additional measurements performed on TSP–paper composites and on casted specimens can only serve as a qualitative reference for the data obtainedfor the airbrushed TSP layer specimen. Nevertheless, the results illustrate the differences between properties of a thin layer and bulk material structures.

The results of microcalorimetric measurementof the TSP layer revealed a glass transition effect between approximately 30 and 50 °C. The results of additional measurements confirm this. The transition effects do not affect the thermal transport properties of the investigated coatingmuch. The same concerns the moisture sorption effects revealed in the course of repeated thermogravimetric studies—in practical applications of TSP, these effects could be treated as being of minor importance.

## 11. Conclusions

In this paper, we have presented the results of a detailed investigation of the thermal properties of a TSP coating applied by airbrush spraying. The TSP study was complemented with results of an investigation of TSP structures obtained by paint casting. To our surprise, the two methods of TSP layer manufacturing resulted in differences in the layer structures and properties. This observation contributes to a better understanding of the results obtained for the basic, aerosol-sprayed TSP coating. It has also been confirmed that the layer deposition technique plays a crucial role in shaping both its structure and properties.

The TSP layer from aerosol spraying exhibited thermal properties typical for insulators. A similar general conclusion is provided in [21]. However, the present research provides not only corrected out-of-plane TSP thermal diffusivity data but also complements previous outcomes with complex thermophysical property investigation results that include in-plane thermal diffusivity estimation results. The measurement results are burdened with the large uncertainty of the thermal diffusivity estimation. Nevertheless, the correctness of the measurements was confirmed by the compliance of the diffusivity measurement results obtained for samples of different thicknesses. Furthermore, a change in TP characteristics between 20 and 60 °C was also observed and can be included in a detailed analysis of the measurement results—for example, when applying TSP data as the input data for inverse problem solution or in the modeling of an air-TSP heat transfer processmodel. Nevertheless, it should be underlined that the TP changes according to the temperature change are moderate, even at the temperature variation scale indicated in [19,20]. The same concerns sensitivity to atmospheric humidity, whichwas proven to be only moderate. The presented data can be helpful in increasing the accuracy of heat transfer studies with the use of TSP. The measurement procedures of thermal properties’ investigation can be used in different research, e.g., for pressure-sensitive paint [19] property research.

## Figures and Tables

**Table 1:** Transitions selected for thermometry.

Parameter	Description	µm
Ra	Roughness average	10.37
Rq	Root mean square roughness	14.55
Rz(ISO)	Average maximum height of the profile	57.48
Rp	Maximum profile peak height	46.60
Rmax	Maximum roughness depth	74.80
Rv	Maximum profile valley depth	28.20

**Table 2:** Thermal properties of materials in 1D model of heat conduction simulation.

BC: Step Heat Flux, Heat Convection							
Domain–Structure	Material	Thickness, μm		Density, *ρ*, kg·m^−3^	Heat Capacity, *c_p_*, J·kg^−1^·K^−1^		Thermal Conductivity, *λ*, W·m^−1^·K^−1^
		3-Layer TSP	5-Layer TSP		T < 40 °C	T > 40 °C	
Layer 1	Graphite	10	10	780	800	800	1.2
Layer 2	Molybdenum	96	96	9999	251	251	138
Layer 3	TSP	15	30	1980	860	950	Estimated
Layer 4	Graphite	10	10	780	800	800	1.2

**Table 3:** The TSP thermal parameters at room temperature versus the data presented by Cai et al. [15].

Structure	Density, *ρ*	Heat Capacity, *c_p_*	Thermal Conductivity, λ	Thermal Diffusivity, *a*	Comments
	kg·m^−3^	J·kg^−1^·K^−1^	W·m^−1^·K^−1^	mm^2^·s^−1^	
Airbrushed TSP layer	1980	890	0.58	0.33	TC, TD—out of plane
Casted (dried) TSP	1380	1270	−−	−−	−−
TSP–paper composite	1420	−−	−−	0.34	Effective, in plane
20-μm TSP layer—Cai et al.	1300	1090	0.15	0.106	−−

## Data Availability

Data is contained within the article.

## Nomenclature

*a*	Thermal diffusivity
*c_p_*	Specific heat (material)/heat capacity (structure)
*T*	Temperature
*λ*	Thermal conductivity
*ρ*	Density
**Abbreviations**	
CLTE	Coefficient of linear thermal expansion
DSC	Differential scanning calorimetry
IR	Infrared (imaging)
LFA	Laser flash apparatus/analysis
SEM	Scanning electron microscopy
TC	Thermal conductivity
TG	Thermal diffusivity
TG	Thermogravimetry/-ic
TP	thermophysical property/-ies
TSP	Temperature-sensitive paint
